# Population structure and breed identification of Chinese indigenous sheep breeds using whole genome SNPs and InDels

**DOI:** 10.1186/s12711-024-00927-1

**Published:** 2024-09-03

**Authors:** Chang-heng Zhao, Dan Wang, Cheng Yang, Yan Chen, Jun Teng, Xin-yi Zhang, Zhi Cao, Xian-ming Wei, Chao Ning, Qi-en Yang, Wen-fa Lv, Qin Zhang

**Affiliations:** 1 Shandong Provincial Key Laboratory for Livestock Germplasm Innovation & Utilization, College of Animal Science, Shandong Agricultural University, Tai’an, 271018 China; 2 CAS Key Laboratory of Adaptation and Evolution of Plateau Biota, Qinghai Key Laboratory of Animal Ecological Genomics, Northwest Institute of Plateau Biology, Chinese Academy of Sciences, Xining, 810001 Qinghai China; 3 Key Lab of Animal Production, Product Quality and Security, Ministry of Education, Jilin Agricultural University, Changchun, 130118 China

## Abstract

**Background:**

Accurate breed identification is essential for the conservation and sustainable use of indigenous farm animal genetic resources. In this study, we evaluated the phylogenetic relationships and genomic breed compositions of 13 sheep breeds using SNP and InDel data from whole genome sequencing. The breeds included 11 Chinese indigenous and 2 foreign commercial breeds. We compared different strategies for breed identification with respect to different marker types, i.e. SNPs, InDels, and a combination of SNPs and InDels (named SIs), different breed-informative marker detection methods, and different machine learning classification methods.

**Results:**

Using WGS-based SNPs and InDels, we revealed the phylogenetic relationships between 11 Chinese indigenous and two foreign sheep breeds and quantified their purities through estimated genomic breed compositions. We found that the optimal strategy for identifying these breeds was the combination of DFI_union for breed-informative marker detection, which integrated the methods of Delta, Pairwise Wright's FST, and Informativeness for Assignment (namely DFI) by merging the breed-informative markers derived from the three methods, and KSR for breed assignment, which integrated the methods of K-Nearest Neighbor, Support Vector Machine, and Random Forest (namely KSR) by intersecting their results. Using SI markers improved the identification accuracy compared to using SNPs or InDels alone. We achieved accuracies over 97.5% when using at least the 1000 most breed-informative (MBI) SI markers and even 100% when using 5000 SI markers.

**Conclusions:**

Our results provide not only an important foundation for conservation of these Chinese local sheep breeds, but also general approaches for breed identification of indigenous farm animal breeds.

**Supplementary Information:**

The online version contains supplementary material available at 10.1186/s12711-024-00927-1.

## Background

Sheep (*Ovis aries*), one of the earliest domesticated livestock species, have a widespread distribution in the world, particularly in China. The geographical features of China, including its vast territory, complex topography and significant variations in altitude, contribute to diverse ecological conditions and climate patterns. These factors have had a profound impact on the formation and distribution of domestic animal diversity in China [1]. Through long-term adaptation to local environments, numerous distinctive attributes have been accumulated within local sheep populations. These characteristics encompass high productivity, extensive adaptability, early maturity, and disease resistance. However, due to indiscriminate crossbreeding practices and insufficient conservation efforts, many breeds are declining in population size and facing the risk of extinction. Therefore, there is a pressing need to investigate the existing genetic diversity, and to take efficient measures for better preserving these indigenous sheep breeds.

Efficient and accurate identification of animals belonging to a particular breed is essential for the effective conservation and sustainable use of these local breeds [2]. Up to now, animal population structure and breed identification studies have been conducted mainly based on SNP chip data or whole genome sequencing (WGS) data [3–6]. WGS has the advantage of capturing rare species-specific polymorphisms and more informative polymorphisms compared to SNP chips [7, 8]. In addition, WGS provides the opportunity to capture various additional types of variants besides SNPs across the genome, such as Insertions/deletions (InDels), which are the second most common type of genomic variant, with an estimated ratio of 1 InDel for every 5.3 SNPs [9]. Research conducted on humans has revealed that InDels play prominent roles in evolutionary changes [10, 11]. Therefore, it becomes important to investigate the potential of InDels in the context of population structure analysis and breed identification.

The breed identification procedure typically includes three key steps: (1) establishment of a training population consisting of purebred individuals of multiple breeds, (2) selection of the most breed-informative markers relevant to the breeds in the training population, and (3) assignment of test individuals to a particular breed within the training population. Indigenous breeds have often suffered from gene introgression of other breeds due to unintended or intended crossbreeding. Therefore, it is necessary to examine the purity of animals in the training population. This can be done by estimating the genomic breed compositions (GBC) using genomic data. Many methods have been proposed to estimate GBCs of individuals, such as linear regression models [12, 13], supervised admixture models [14], and SNP-BLUP models [15]. Several studies have shown that the supervised admixture model gave more consistent results, even when the number of SNPs was small [16–18].

A number of methods have been proposed for breed-informative SNP detection and breed assignment. In our previous study [6], using a training population consisting of commercial cattle breeds and SNPs from WGS, we compared three methods for detecting breed informative SNPs, i.e., Delta [19], Pairwise Wright’s F_ST_ [20], and Informativeness for Assignment (*I*_*n*_) [21], as well as five machine learning classification methods for breed assignment, i.e., K-Nearest Neighbor (KNN), Support Vector Machine (SVM), Random Forest (RF), Naïve Bayes, and Artificial Neural Network. Our results demonstrated that the optimal strategy was to use the common SNPs identified by Delta, *F*_ST_, and *I*_n_ and to integrate KNN, SVM, and RF for breed assignment. However, it is not clear whether this strategy is also optimal for breed identification of indigenous breeds.

In this study, we investigated the phylogenetic relationships and genetic purities of 13 sheep breeds using WGS-based SNP and InDel data. The breeds included 11 Chinese indigenous breeds, most of which were facing extinction, and 2 foreign commercial breeds. We compared different strategies for breed identification with respect to different marker types (SNPs, InDels, and a combination of SNPs and InDels), different breed-informative marker detection methods, different machine learning classification methods, and different numbers of markers. We developed an optimal strategy and marker panels for breed identification specific to the breeds involved in this study.

## Methods

### Animals and WGS data

The data used in this study was WGS data of 13 sheep breeds, including 11 Chinese indigenous breeds and two foreign commercial breeds. The WGS data of three Chinese breeds, Guide Black Fur sheep, Minxian Black Fur sheep and Hanzhong sheep were obtained by sequencing their blood samples with the DNBSEQ-T7 platform. The WGS data of other breeds were downloaded from the National Center for Biotechnology Information (NCBI) databases (https://www.ncbi.nlm.nih.gov/). A full description of the samples is detailed in Additional file 1: Table S1. The number of individuals of each breed ranged from 18 to 39, with a total of 349 individuals. The breed names, numbers of individuals, and average sequencing depths of the 13 breeds are presented in Table 1.

**Table 1:** Sheep breeds involved in this study and their average sequencing depths (± SD), total numbers of animals, and numbers of most-likely purebred animals

Breed name	Code	Average sequencing depth (X)	Total number of animals	Number of most-likely purebred animals
Chinese indigenous breeds				
Bashbay	BSB	17.63 ± 10.00	29	24
Altay	ALT	14.73 ± 8.47	26	26
Cele Black	CLB	17.41 ± 9.49	29	23
Duolang	DL	17.12 ± 9.79	34	28
Tan sheep	TAN	19.72 ± 12.11	23	19
Hu sheep	HU	15.98 ± 10.80	30	29
Wadi sheep	WD	40.48 ± 4.50	20	20
Small-Tailed Han	STH	18.75 ± 13.24	22	22
Minxian Black Fur	MBF	46.55 ± 6.56	30	20
Guide Black Fur	GBF	42.07 ± 7.08	30	29
Hanzhong	HZ	42.14 ± 4.46	39	39
Foreign breeds				
Australian Merino	AMR	21.30 ± 3.98	18	17
Dorset	DS	14.55 ± 2.60	19	18
Total		26.07 ± 14.94	349	314

### SNPs/InDels calling and quality control

We removed adapters and trimmed low-quality ends from raw reads using Fastp v0.23.2 [22]. Sentieon’s DNASeq pipeline (https://www.sentieon.com/products/) was used to call SNPs and InDels as follows: (i) the functions “bwa mem”, “util sort” and “Dedup” were used to map clean reads to the sheep reference genome [23], sort bam files and remove duplicates, (ii) raw GVCFs were called from the bam files using the function “Haplotyper”, and (iii) the individual GVCFs were merged and called jointly to generate VCFs using the function “GVCFtyper”. To avoid potential false-positive calls, we used the “VariantFiltration” function of GATK v4.2.6.1 [24] to filter SNPs with the following criteria parameters: QD (quality by depth) < 2.0, MQ (mapping quality) < 40.0, FS (Fisher strand) > 60.0, SOR (strand odds ratio) > 3.0, MQRankSum (mapping quality rank sum test) < − 12.5, and ReadPosRankSum (read position rank sum test) < − 8.0. For InDel filtering, the criteria were QD < 2.0, FS > 200.0, SOR > 10.0, MQRankSum < − 12.5, and ReadPosRankSum < − 8.0. We obtained 60,554,004 SNPs and 6,995,733 InDels.

Quality control of the three types of genotype data, i.e., SNPs, InDels, and SIs, were carried out using Plink v1.9 [25]. SNPs/InDels/SIs were removed if the following requirements were not met: (i) being biallelic, (ii) 100% genotyping rate (several methods used in this study for detection of breed-informative SNPs or classification do not allow any missing values), (iii) locating on autosomes, and (iv) InDels < 50 bp. After quality control, 17,623,634 SNPs and 1,539,027 InDels were retained. Further, we pruned SNPs/InDels with linkage disequilibrium (LD) *r*^2^ ≥ 0.2 within a 500-kb window, so that SNPs/InDels with stronger LD than *r*^2^ ≥ 0.2 were removed. Finally, we obtained 822,488 SNPs and 464,035 InDels. In addition to using these two types of markers separately, we also used SI markers. After pruning SIs using the same LD *r*^2^ threshold of 0.2 within a 500-kb window, we obtained 857,010 SIs (including 774,180 SNPs and 82,830 InDels).

### Population structure analysis

Based on the 822,488 SNPs, the phylogenetic tree analysis was performed to estimate the phylogenetic relationships among all 349 individuals. The VCF2Dis software (https://github.com/BGI-shenzhen/VCF2Dis) was used, and the results were then utilized as input for FastMe2.0 [26] to generate a Neighbor-Joining (NJ) phylogenetic tree. The exported phylogenetic tree was visualized using the iTOL program (https://itol.embl.de/). Individuals that were grouped in a breed other than their labeled breed were removed from the dataset and the remaining individuals were used for subsequent analyses.

### Estimation of genomic breed composition

A supervised admixture analysis using Admixture v1.3 [27] was performed to estimate GBC of each individual. A tenfold cross validation was carried out, in which each breed was randomly divided into ten subsets, of which nine were used in turn as the reference group and the remaining one was treated as the test group.

### Establishment of the training population

A training population for breed identification should consist of purebred animals of relevant breeds. Therefore, it is necessary to identify purebred animals of each breed. Based on the results of population structure analysis and GBC estimation, we identified an individual as most-likely purebred of a breed if it was within the branch of that breed in the phylogenetic tree and had an estimated GBC of that breed ≥ 90%. Only individuals identified as most-likely purebred were included in the training population.

### Detection of breed informative markers

We used five different strategies to detect breed-informative markers (SNPs, InDels, or SIs), namely DFI_inter, DFI_union, MDA, MDG, and MI.

#### DFI_inter and DFI_union

In our previous study, we proposed the DFI method [6], which is a combination of three methods, i.e., Delta [19], Pairwise Wright’s F_ST_ [20] and Informativeness for Assignment [21], in which the common markers derived from the three methods are extracted as the final markers. This method is named DFI_inter in this study. An alternative way to combine the three methods is to select equal number of top breed informative markers from the three methods and merge them together to achieve the defined total number of unique markers. This method is named DFI_union.

#### MDA and MDG

The RF algorithm is a popular machine learning technique used for classification and regression tasks. It can rank predictors based on correlations observed within classification rules, providing two relevance measures: the mean decreased accuracy (MDA) and the mean decreased Gini index (MDG). These two ranking rules can be utilized to assess the SNPs present in the dataset, thereby allowing the identification of a crucial set of discriminatory markers [28, 29]. The randomForest function of R package randomForest [30] with default parameters was used to obtain the breed informative markers measured by MDA and MDG.

#### Mutual information

Mutual information (MI) is a filtering method that ranks the SNPs according to their pairwise relevance to the breed label. Because it assesses each feature individually, it may fail to discover important feature groups and choose redundantly correlated ones. Nevertheless, due to its low computational complexity, it is still popular when dealing with high-dimensional datasets in bioinformatics studies [31, 32]. The mutinformation function of R package infotheo [33] with default parameters was used to perform the MI method.

### Classification methods for breed assignment

Four machine learning models (KNN, SVM, RF and KSR) were used to classify individuals for breed assignment. We used the most breed-informative SNPs/Indels/SIs identified from the training population to train the models by aligning the markers of individuals in the test population with the most breed-informative (MBI) markers of individuals in the training population. Different numbers of MBI markers were considered: 200, 400, 600, 800, 1000, 2000, 3000, 4000, 5000, and 6000. The larger markers sets included the smaller marker sets.

K-Nearest Neighbor (KNN) is a supervised machine learning algorithm that classifies new samples based on their proximity to the training set. Specifically, for an unclassified sample, KNN identifies its closest neighbors k in the training set and assigns a class label by taking the majority vote of the class labels among these neighbors [34]. The knn function of R package class [35] with default parameters was used to perform KNN classification.

Support Vector Machine (SVM) achieves classification by maximizing the margin between the optimal hyperplane (the decision boundary for breed classification) and the nearest samples of different classes, while minimizing the classification error on the training set [36]. The svm function of R package e1071 [37] (with the option type = C-classification, kernel = linear) was used to perform SVM classification.

Random Forest (RF) achieves classification by building an ensemble of decision trees, where each tree is constructed using a random subset of features and a bootstrapped sample from the training set. The final class label is determined by aggregating the predictions of all individual trees through majority voting or weighted voting [38]. The randomForest function of R package randomForest [30] with default parameters was used to perform RF classification.

KSR integrates the three methods (KNN, SVM and RF) by taking the intersection of their results, i.e., the intersection of at least two of the three methods. If there was no intersection at all, we took the result of KNN [6].

For each of these methods, 50 replicates were carried out with different random sampling procedures within the machine learning framework.

### Breed identification

Breed identification was performed using the aformentioned breed-informative marker detection methods, machine learning classification methods with different MBI markers, and marker types. A tenfold cross validation was carried out to evaluate the accuracy of breed identification under different scenarios. Each breed in the established training population was divided into 10 subsets of the same size, one of which in turn was used as validation set and the remaining nine as training sets.

The accuracy of breed identification was defined as follows:

$$ Accuracy = \frac{1}{50}\mathop \sum \limits_{i = 1}^{50} \frac{{N_{T} }}{{N_{T} + N_{F} }} \times 100\% $$

where ${N}_{T}$ is number of individuals which were correctly assigned to the breeds they belong to and ${N}_{F}$ is the number of individuals which were wrongly assigned.

## Results

### Population structure

**Fig. 1:**
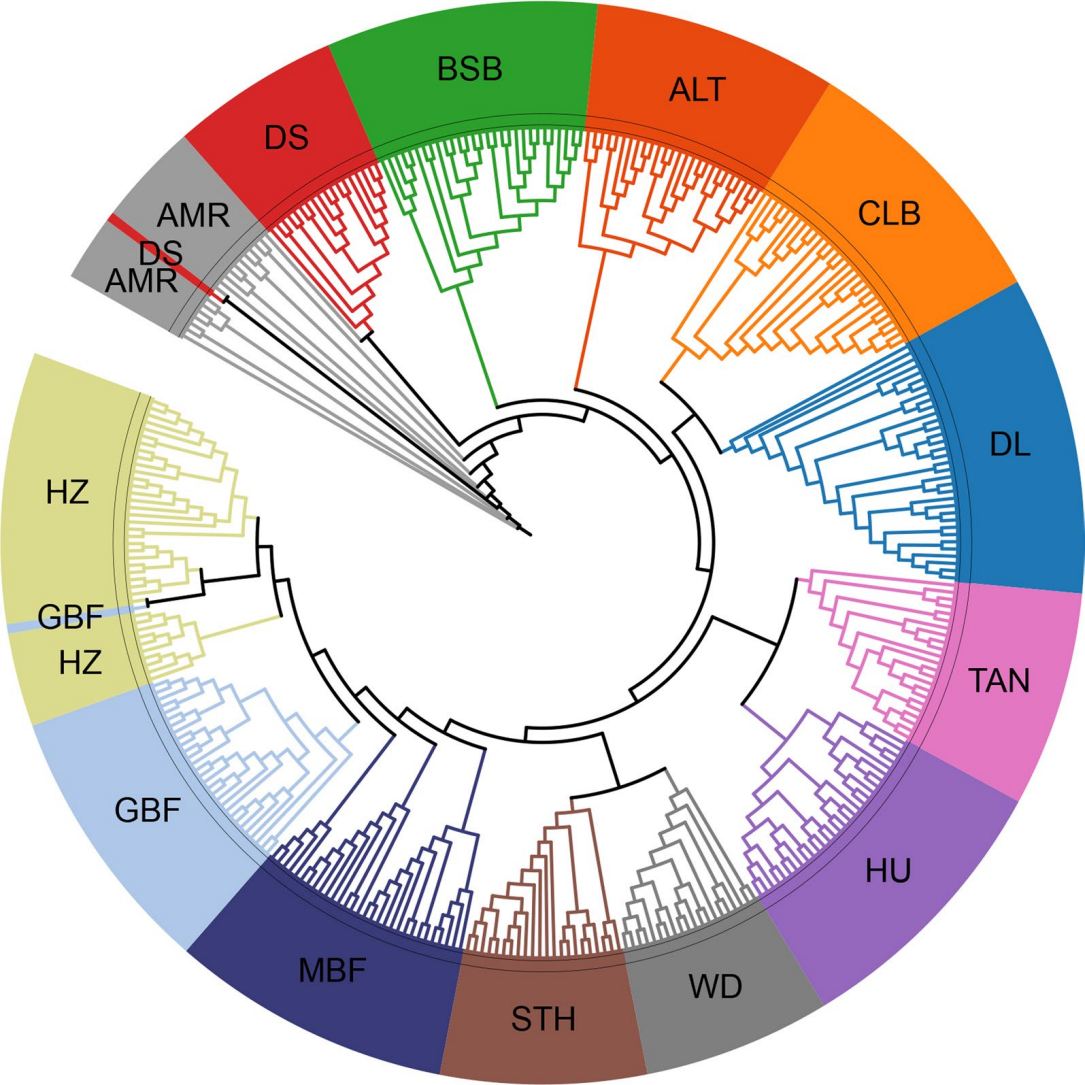
Phylogenetic relationships between 13 sheep breeds revealed using 822,488 SNPs. The breed names represented by the codes for different colors are presented in Table 1

The phylogenetic tree showed the relationships between the 13 breeds as well as between individuals within each breed. The 349 individuals were grouped into 13 branches corresponding to the 13 breeds (Fig. 1). However, two individuals, which had breed labels DS and GBF, were grouped in branches AMR and HZ, respectively. These two individuals were removed from the dataset.

### GBC estimation

**Fig. 2:**
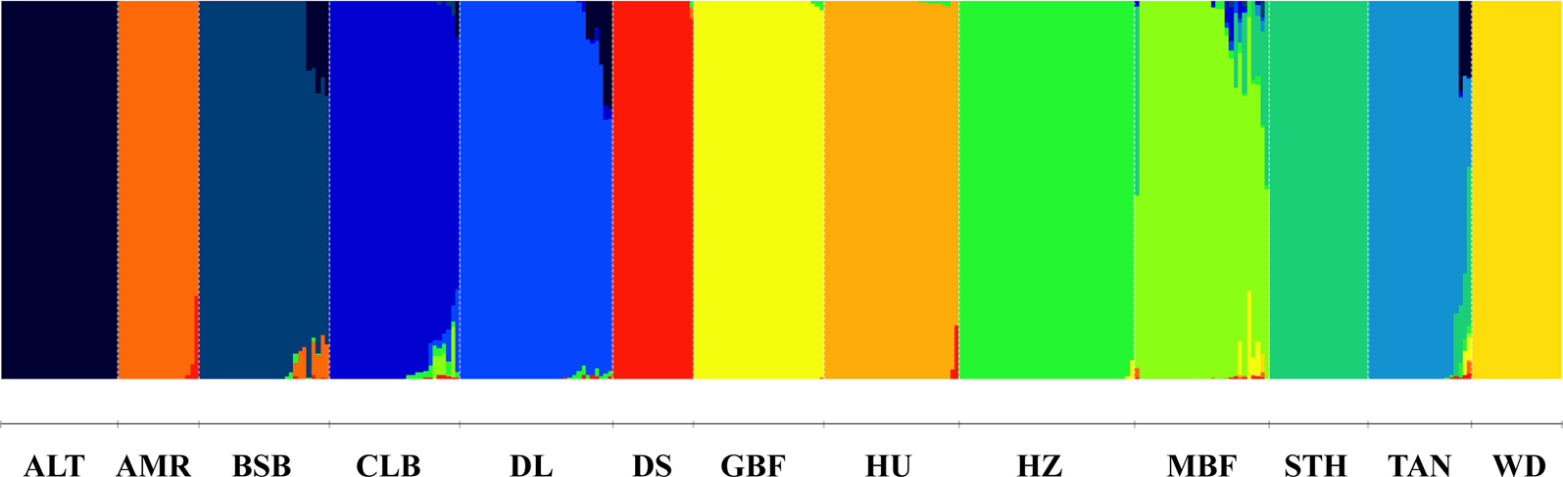
Genomic breed compositions estimated using supervised admixture analysis. The breed names represented by the codes for different colors are presented in Table 1

The GBC estimates of all individuals obtained using a supervised admixture analysis with 822,488 SNPs are illustrated in Fig. 2. In breeds BSB, CLB, DL, HU, MBF, and TAN there were some individuals that had small GBCs for two or more other breeds. For each breed, the average GBCs of the 13 breeds are presented in Table 2.

**Table 2:** Average GBCs (%) of different breeds in each breed (in rows) estimated using 822,488 SNPs

Breed*	ALT	BSB	CLB	DL	TAN	STH	HZ	MBF	GBF	WD	HU	AMR	DS
ALT	*99.99*	0.00	0.00	0.00	0.00	0.00	0.00	0.00	0.00	0.00	0.00	0.00	0.00
BSB	3.66	*94.06*	0.00	0.00	0.00	0.00	0.26	0.00	0.00	0.00	0.00	1.92	0.08
CLB	0.34	0.24	*95.74*	1.61	0.00	0.00	0.85	1.04	0.00	0.00	0.00	0.00	0.18
DL	2.98	0.00	0.43	*96.07*	0.00	0.00	0.40	0.00	0.00	0.00	0.00	0.00	0.12
TAN	2.79	0.00	0.08	0.00	*91.80*	3.95	0.44	0.04	0.55	0.00	0.02	0.14	0.19
STH	0.00	0.00	0.00	0.00	0.00	*99.99*	0.00	0.00	0.00	0.00	0.00	0.00	0.00
HZ	0.00	0.00	0.00	0.00	0.00	0.00	*99.84*	0.00	0.15	0.00	0.00	0.00	0.00
MBF	0.00	0.25	0.85	0.33	0.37	6.71	0.73	*88.73*	1.53	0.16	0.03	0.16	0.16
GBF	0.00	0.00	0.00	0.00	0.00	0.00	0.17	0.00	*99.81*	0.00	0.00	0.00	0.01
WD	0.00	0.00	0.00	0.00	0.00	0.00	0.00	0.00	0.00	*99.99*	0.00	0.00	0.00
HU	0.00	0.00	0.00	0.00	0.00	0.00	0.20	0.00	0.00	0.00	*99.22*	0.00	0.57
AMR	0.00	0.00	0.00	0.00	0.00	0.00	0.00	0.00	0.00	0.00	0.00	*98.48*	1.51
DS	0.00	0.00	0.00	0.00	0.00	0.00	0.11	0.00	0.00	0.00	0.00	0.16	*99.73*

^*^ The breed names represented by these codes are presented in Table 1

Italic values are the average GBC of the breed for individuals which were labeled as that breed

We treated individuals with ≥ 90% GBC of their corresponding breed as most-likely purebred animals. These individuals (314 in total, Table 1) were included in the training population for breed identification.

**Fig. 3:**
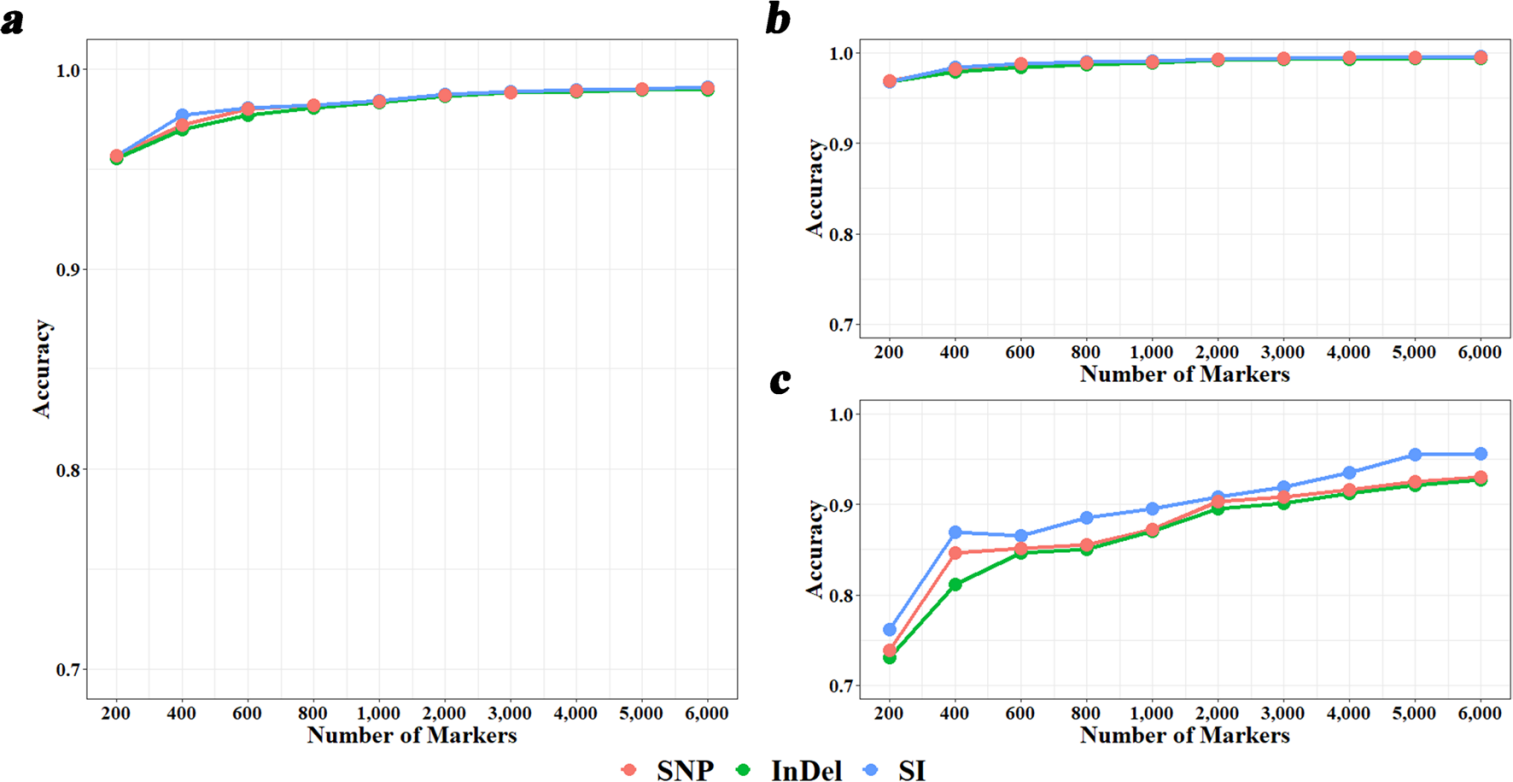
Accuracies of genomic breed composition (GBC) estimation using different numbers of most breed-information SNPs, InDels, and SIs revealed by DFI_union. **a** Overall correlations between GBCs using different numbers of most breed-information markers and GBCs using 822,488 SNPs for all of the 347 individuals. **b** Correlations for the 314 most-likely purebred individuals. **c** Correlations for the rest 33 individuals

We also estimated GBCs using different numbers of the most breed-informative SNPs, InDels, or SIs based on the previously described selection methods and looked at their accuracies in GBC estimation, measured as correlations between these GBCs and those from using all 822,488 SNPs. Overall, the accuracies were very high with correlations over 0.95 (Fig. 3a) and the three marker types performed very similar. In particular, for the most- likely purebred individuals defined above, the correlations were all > 0.96 (Fig. 3b). However, for the other individuals, which were likely non-purebred animals, the accuracies were much lower with correlations ranging from 0.73 to 0.93 for different number of SNPs or InDels and ranging from 0.76 to 0.96 for different number of SIs (Fig. 3c). SI markers performed better in this context.

### Accuracies of breed identification using SNPs

**Fig. 4:**
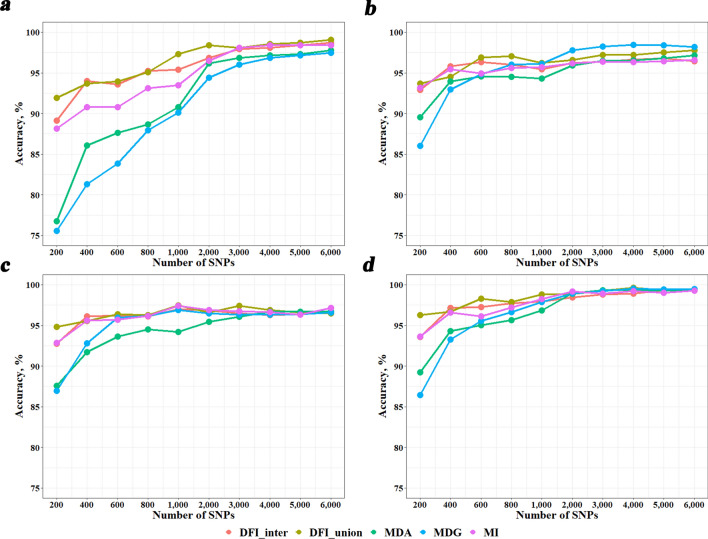
Breed identification accuracies using different numbers of most breed-informative SNPs under different scenarios. **a**–**c** and d refer to machine learning classification methods KNN, RF, SVM and KSR, respectively. *KNN* K-Nearest Neighbor; *RF* Random Forest; *SVM* Support Vector Machine; *KSR* an integration of KNN, SVM and RF

Figure 4 shows the breed identification accuracies of five breed-informative marker detection methods (MDA, MDG, MI, DFI_inter, and DFI_union), different machine learning classification methods (KNN, RF, SVM, and KSR), and different numbers of most breed-informative SNPs (200–6000). When the number of SNPs exceeded 1000, the accuracies were over 90% in all scenarios. The KSR classification method performed the best with accuracies over 95% when the numbers of SNPs were over 1000 and over 99% when the numbers of SNPs were over 2000. The method was very robust with respect to different breed-informative marker detection methods and different numbers of SNPs. The performances of the other three classification methods were significantly affected by the marker detection methods and the number of SNPs, with no priority of one over the other method. In general, the accuracies increased with the increase of the number of SNPs, but became stable when the number of SNPs was greater than 4000. Different breed-informative marker detection methods performed differently in different classification methods, e.g., for the RF method, MDG performed best, while for the KNN method, MDG was the worst. DFI_union performed better in most cases although it was not the best for the RF method.

### Comparison of breed identification accuracies using SNPs, InDels, or SIs

**Fig. 5:**
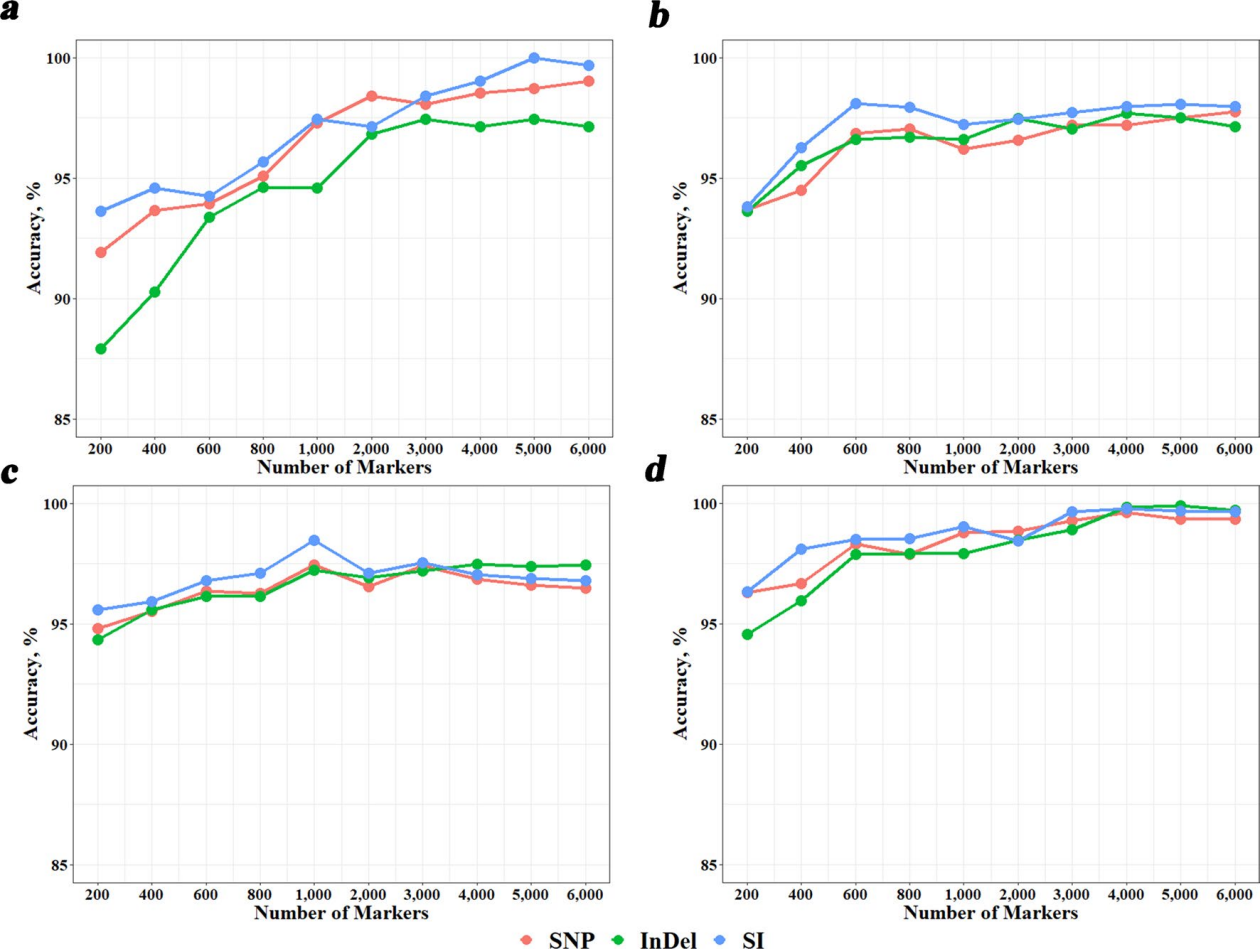
Breed identification accuracies using three different types of most breed-informative markers revealed using DFI_union. **a**–**d** refer to machine learning classification methods KNN, RF, SVM and KSR, respectively. *KNN* K-Nearest Neighbor; *RF* Random Forest; *SVM* Support Vector Machine; *KSR* an integration of KNN, SVM and RF

Figure 5 shows the breed identification accuracies using the three marker types under four different classification methods. Here, only the DFI_union method was utilized to detect breed-informative markers. In most cases, using SIs yielded better accuracies than using SNPs or InDels, and achieved nearly accuracies of 100% in some cases. For KNN, using SNPs was generally better than using InDels, while for the other methods, there was no priority between using SNPs and InDels. Again, KSR performed best and gave accuracies over 97.5% when the number of SNPs was greater than 600, and was robust to marker types.

Table 3 presents the detailed accuracies for each breed by different classification methods using 5000 SIs revealed by DFI_union. For seven breeds, namely CLB, DL, GBF, HU, HZ, AMR, and DS, accuracies of 100% were obtained by all classification algorithms. For the other breeds, KNN and KSR still yielded all accuracies of 100% except for MBF with an accuracy of 95.3% by KSR. RF and SVM yielded all accuracies over 95%, except for breeds MBF and TAN, where accuracies lower than 85% were obtained. Further detailed analysis revealed that, for MBF, the misclassified individuals were primarily assigned to breeds STH or GBF, and for TAN, the misclassified individuals were primarily assigned to ALT (Additional file 1 Table S2 and Table S3).

**Table 3:** Average identification accuracies (%) in different breeds by different machine learning methods using 5000 most breed-informative SIs revealed by DFI_union

Breed^a^	No Anim	Classification methods^b^			
		KNN	RF	SVM	KSR
ALT	26	100.00	99.38	99.85	100.00
BSB	24	100.00	97.50	99.00	100.00
CLB	23	100.00	100.00	100.00	100.00
DL	28	100.00	100.00	100.00	100.00
GBF	29	100.00	100.00	100.00	100.00
HU	29	100.00	100.00	100.00	100.00
HZ	39	100.00	100.00	100.00	100.00
MBF	20	100.00	83.00	73.50	95.30
STH	22	100.00	96.64	97.27	100.00
TAN	19	100.00	98.95	81.58	100.00
WD	20	100.00	95.20	99.70	100.00
AMR	17	100.00	100.00	100.00	100.00
DS	18	100.00	100.00	100.00	100.00
Total	314	100.00	98.07	96.90	99.70

^a^The breed names represented by these codes are presented in Table 1

^b^*KNN* K-Nearest Neighbor; *RF* Random Forest; *SVM* Support Vector Machine; *KSR* an integration of KNN, SVM, and RF

## Discussion

Establishing a training population is fundamental for developing methods and a marker panel for breed identification. The training population should consist of purebred individuals of relevant breeds. To achieve this, we first conducted a phylogenetic tree analysis. The results show that the studied individuals from 13 breeds were well grouped into 13 branches corresponding to the 13 breeds with only two individuals that were grouped into branches different from their labeled breeds. The most likely reason is a wrong breed labelling. The phylogenetic relationships between these breeds are consistent with their origins. The 11 Chinese indigenous breeds have three different origins. The breeds BSB, ALT, CLB, and DL originated from the Kazakh sheep; TAN, HU, WD, and STH originated from the Mongolian sheep; and MBF, GBF, and HZ originated from the Tibetan sheep [1, 39]. The two foreign breeds, AMR and DS, originated from Australia. All breeds of the same origin were adjacent to each other in the phylogenetic tree. Then, we evaluated the breed purity of each individual by estimating its GBCs using a supervised admixture analysis. In some studies, an unsupervised admixture analysis was used for GBC estimation. However, several studies have shown that using a supervised admixture analysis is more appropriate for labeled data [14, 40], which is the case in our study. In addition, it was shown that an unsupervised analysis was not suitable for the case where the sample sizes of different breeds in the training population are unbalanced [40], which is also the case for our study, i.e., the sample sizes ranged from 17 to 39. We also tried using an unsupervised analysis, and it turned out that the optimal K value was not 13 (number of breeds), but rather 3. This is unreasonable. Therefore, we did not use an unsupervised admixture analysis to estimate GBCs.

Breed identification has been conducted overwhelmingly using SNPs genotyped with a SNP chip or sequencing [3–6, 41]. Whole genome sequencing has the advantage of providing not only SNP genotypes but also other types of genomic variants, such as InDels, which are the second most common type of genomic variants following SNPs [9]. We were interested in whether InDels are better markers than SNPs for breed identification, or using SNPs and InDels together can improve the accuracy of breed identification compared to just using SNPs or InDels. We observed that when using KNN as the classification method, SNPs performed consistently better than InDels, while there was no difference between using SNPs and InDels when using the other classification methods. However, the differences were generally small. Using SNPs and InDels together (i.e., SIs) could improve the accuracies in most cases, although the improvement was small, because the accuracies from SNPs or InDels were already very high. In addition, the SI markers have an advantage in GBC estimation for the likely non-purebred individuals (Fig. 3c).

The accuracy of breed identification is contingent upon not only the marker selection methods and classification methods, but also the training population, i.e., breed purity, genetic relatedness between breeds, and sample size of each breed within the training set. In this study, all the identification errors occurred in breeds that all had relatively small sample sizes (less than 25), and/or close phylogenetic relationships with breed(s) to which the wrongly identified individual(s) were assigned. This is particularly the case for MBF, which had the highest identification error rate. MBF had a sample size of 20 (Table 1) and very close phylogenetic relationships with GBF and STH (Fig. 1), and almost all misclassified individuals were assigned to breeds STH or GBF. We noticed that the two foreign breeds (AMR and DS) had the smallest sample sizes (17 and 18, respectively), but no identification error occurred for them. This is most likely due to their distant phylogenetic relationships with other breeds in this dataset.

Exploring the most effective strategies for detecting breed-informative markers and machine learning classification methods holds significant value for breed identification. In our previous study on commercial cattle breeds [6], we found that the breed-informative marker detection method DFI (named DFI_inter in this study), combined with the classification method KSR, was the optimal strategy because it produced the best accuracy in most cases and was very robust to various conditions, e.g., training population size, and number of SNPs. In this study, we further compared additional breed-informative marker detection methods, DFI_union, MDA, MDG, and MI. Unlike DFI_inter, DFI_union selects breed-informative markers by merging the markers from the three methods, instead of taking the common markers from the three methods. For DFI_inter, we needed more markers from each method to obtain a common panel with the required number of markers. For example, we found that to obtain a certain number of common SNPs, we needed more than twice that number of SNPs from each method. Thus, the final marker panel may contain many less informative markers of each method. This may decrease the accuracy of breed identification. Indeed, our results showed that DFI_union performed better than DFI_inter in almost all scenarios, and also the best among all methods in most scenarios, with different machine learning classification methods and number of markers. The same as in our previous study, we found that the KSR classification method was very robust and performed best with an identification accuracy over 97.5% when the number of SNPs was greater than 1000 (Figs. 4 and 5).

One of the goals of this study is to develop a panel of markers that can be used for breed identification specifically for the Chinese sheep breeds investigated in this study. It should be noted that breed identification can also be conducted using an existing commercial SNP chip. The advantages of using the panel (with 1000–5000 markers) we developed over using a commercial SNP chip (with over 50,000 markers) are (1) these markers were selected from the sequence data of the relevant breeds and thus are more breed-informative specifically for these breeds. Using these markers would achieve higher accuracy of breed identification than using a commercial ship which was developed based on sequence data of some commercial breeds; (2) it is much cheaper for genotyping; and (3) it is computationally much easier.

## Conclusions

By comparing different breed-informative marker detection methods and machine learning classification methods, we proposed an efficient general approach for breed identification. Based on this approach, we developed a marker panel consisting of ~ 1000 to 5000 of the most breed-informative markers specifically for the sheep breeds investigated in this study. Compared to a commercial SNP chip, this panel provides a more cost-effective and accurate tool for breed identification, which will contribute to improving the effective conservation and sustainable utilization of Chinese indigenous sheep breeds.

### Supplementary Information

Additional file 1. **Table S1.** Detailed sequencing information of the 349 individuals involved in this study. **Table S2.** Average (over 50 replicates) numbers of individuals assigned to different breeds in each breed (in rows) based on the random forest classification algorithm using 5000 most breed-informative SIs revealed by DFI_union. **Table S3.** Average (over 50 replicates) numbers of individuals assigned to different breeds in each breed (in rows) based on the support vector machine classification algorithm using the 5000 most breed-informative SIs revealed by DFI_union.

## Data Availability

The raw sequence data for a subset of animals (n = 243) used in this study were downloaded from NCBI (see Additional file 1 Table S1). The sequence data for the remaining animals (n = 106) are available upon request for research purposes.
